# Management of patients with multidrug-resistant organisms in rehabilitation facilities. Results of a survey in the Rhine-Main region, Germany, 2019

**DOI:** 10.3205/dgkh000350

**Published:** 2020-07-03

**Authors:** Ursel Heudorf, Marlene Berres, Sabine Hofmann, Katrin Steul

**Affiliations:** 1 MDRO Network Rhine-Main, Frankfurt/Main, Germany; 2 Public Health Authority Frankfurt am Main, Frankfurt/Main, Germany; 3 MEDIAN Clinic Hessen GmbH & Co. KG, Bad Nauheim, Germany

**Keywords:** multidrug-resistant organisms MDRO, methicillin-resistant Staphylococcus aureus MRSA, multidrug-resistant gram-negative pathogens MRGN, vancomycin-resistant enterococci VRE, rehabilitation, hygiene management

## Abstract

**Introduction:** Multidrug-resistant organisms (MDRO) are a problem in medical facilities, including rehabilitation facilities in Germany. The national recommendations of the Commission for Hospital Hygiene and Infection Prevention (KRINKO) for prevention of and dealing with patients affected by MDRO are obligatory in rehabilitation facilities. A survey on the management of patients with MDRO in rehabilitation facilities in the Rhine-Main area is presented below.

**Materials and methods:** The questions from a recently published survey in 45 rehabilitation facilities in 26 European countries (Doherty et al., 2019) were largely adopted unchanged: the type, size, and organization of the facility, availability of guidelines on MDRO, screening and (estimated) prevalence of MDRO, as well as special hygiene measures or restrictions for patients with MDRO.

**Results:** 22 of the 43 institutions contacted participated (58%). All facilities had specific recommendations on how to deal with MDRO and more than 95% had adequate hygiene staff. The facilities encompassed 4,369 beds, with 3,909 (89%) of them in single-bed rooms, and only a few offered 3-bed rooms. About 20% of patients in general rehabilitation and 100% in early neurological rehabilitation are screened on admission. Six (27%) facilities refused to accept patients with MDRO. 40% of the facilities treated these patients in their own room and/or in a separate area. 27% of the facilities prohibited eating in the dining room and participating in hydrotherapy. Only 6 (27%) of the rehabilitation centers indicated that patients with MDRO are allowed to participate in full rehabilitation programs.

**Discussion:** In accordance with the results of Doherty et al. (2019), there were many restrictions for rehabilitation patients with MDRO, indicating considerable need for improvement. Necessary hygiene recommendations to avoid the transmission of MDRO must not lead to rejection of inpatient rehabilitation or to less intensive rehabilitation.

## Zusammenfassung

**Einleitung:** Multiresistente Erreger (MRE) sind ein Problem auch in Rehabilitationseinrichtungen in Deutschland. Die entsprechenden nationalen Empfehlungen der Kommission für Krankenhaushygiene und Infektionsprävention (KRINKO) zur Prävention von und zum Umgang mit Patienten mit MRE sind auch in Rehabilitationseinrichtungen zu beachten. Nachfolgend wird eine Umfrage zum Management von Patienten mit MRE in Rehabilitationseinrichtungen im Rhein-Main Gebiet vorgestellt.

**Material und Methode:** Die Fragen einer kürzlich publizierten Umfrage in 45 Rehabilitationseinrichtungen in 26 Ländern in Europa (Doherty et al., 2019) wurden weitgehend unverändert übernommen: erfragt wurden Größe, Art und Organisation der Einrichtung, Vorhandensein von Leitlinien zu MRE, Screening und (geschätzte) Prävalenz von MRE, besondere Hygienemaßnahmen bzw. Einschränkungen für Patienten mit MRE.

**Ergebnisse:** 22 der 43 angeschriebenen Einrichtungen nahmen teil (58%). Alle Einrichtungen hatten spezifische Empfehlungen zum Umgang mit MRE und mehr als 95% verfügten über eine angemessene Ausstattung mit Hygienefachpersonal. Die Einrichtungen umfassten 4.369 Betten, 3.909 (89%) davon in Einzelbettzimmern, nur wenige Häuser boten 3-Bett-Zimmer an. Etwa 20% der Patienten in der allgemeinen Rehabilitation und 100% in der neurologischen Frührehabilitation werden bei Aufnahme gescreent. Sechs (27%) Einrichtungen verweigerten die Aufnahme von Patienten mit MREs. 40% der Einrichtungen therapierten die Patienten im eigenen Zimmer und/oder in einem eigenen Bereich für MRE-Patienten. 27% der Einrichtungen verboten die Einnahme der Mahlzeiten im Speisesaal und die Teilnahme an einer Hydrotherapie. Nur 6 (27%) der Reha-Einrichtungen gaben an, dass Patienten mit MRE an einem vollständigen Rehabilitationsprogramm teilnehmen dürfen.

**Diskussion:** In Übereinstimmung mit den Ergebnissen von Doherty et al. (2019) ergaben sich Einschränkungen für Rehabilitationspatienten mit MRE, so dass erheblicher Verbesserungsbedarf besteht. Notwendige Hygieneempfehlungen zur Vermeidung der Übertragung von MRE dürfen nicht zur Ablehnung der stationären Rehabilitation oder zu einer weniger intensiven Rehabilitation führen.

## Introduction

Multidrug resistant organisms (MDRO) are a severe problem in the health-care setting, not only in hospitals, but also in long-term care and rehabilitation facilities [1], [2], [3], [4], [5], [6], [7], [8], [9], [10], [11], [12], [13], [14], [15], [16]. As hospitals often complained that rehabilitation units refused to admit patients colonized or infected with MDRO, one of the three main goals of the network on MDRO Rhine-Main, founded in 2010, is the improvement of the rehabilitation of patients with MDRO [17]. In 2012, the network established an exemplary hygiene plan for rehabilitation units, encompassing recommendations for various MDRO (methicillin-resistant *Staphylococcus* *aureus* [MRSA], vancomycin-resistant Enterococci [VRE], Enterobacteriaceae with extended spectrum beta-lactamases [ESBL]) [18]. In 2014, the German Commission on Hospital Hygiene and Infection Prevention [19] launched its guideline for MRSA. In this guideline, general recommendations for the management of MRSA in hospitals are outlined (screening, hygienic procedures, including isolation of patients colonized with MRSA) and supplemented with special recommendations for rehabilitation facilities. According to KRINKO, every rehabilitation facility should define its risk profile and determine the respective preventive measures, ensuring the best possible compromise between the prevention of MRSA transmissions and the possibility of participating in rehabilitation measures through the appropriate design of processes [19]. Further guidelines were published on the management of multidrug-resistant Gram-negative bacteriae (MRGN) and drug-resistant enterococci, especially vancomycin-resistant enterococci (VRE) [20], [21].

When a European survey of management of patients with MDRO in rehabilitation facilities was published in 2019 [22], the MDRO Rhine-Main network decided to conduct this survey – with some amendments – in the rehabilitation facilities of the Rhine-Main region, Germany. The aims of the survey were 1. to describe the management of patients with MDRO in the Rhine-Main region, and 2. to compare the data with the European survey, which was conducted in 2016, encompassing 45 facilities in 28 European countries.

## Methods

The European Survey’s questionnaire was published as additional material to the article by Doherty et al. [22], encompassing questions on the type and the organization of the respective facility, the number of rooms and beds, the availability of guidelines for the management of patients with MDRO, the screening for and the prevalence of MDRO, the grouping or cohorting of patients with MDRO, and any restriction of the activities of patients with MDRO. This questionnaire was translated into German, with some questions altered. For example: when the European questionnaire asked for the availability of a microbiologist or infectious disease physician in the facility, this question was changed to asking about the hygiene personnel (i.e., hygienists and authorized hygiene practitioners, infection control nurses, authorized hygiene care nurses), which must be available in such an institution according to the German KRINKO guideline on hygiene expert staff [23], [24]. The questions regarding screening and prevalence of MDRO were to be answered separately by general rehabilitation facilities and those with sections for neurological early rehabilitation. The questionnaires were distributed to 43 rehabilitation facilities known to the network in November 2019; the answers were obtained in January 2020 (for the complete questionnaire, see Attachment 1). 

## Results

Twenty-two rehabilitation facilities took part in this survey, of which four had sections for neurological rehabilitation. Twenty (91%) of them were independent facilities with a doctor available 24/7, and two (9%) were attached to an acute care facility. In all of them, guidelines for the management of patients with MDRO were available, with national guidelines of the KRINKO and regional guidelines being predominant (96% and 82%). Hygienists, authorized hygiene practitioners, infection control nurses, and authorized hygiene-care nurses were available in 91%, 100%, 96%, and 91% of the institutions, respectively (Table 1 (Tab. 1)).

The institutions encompassed 4,369 beds, with 3,909 (89%) of them in single-bed rooms and only a few offering 3-bed rooms. The facilities stated that 58% (range 5–95%) of the patients are admitted directly from acute-care hospitals, 42% (range 3–95%) from their home, and 0.7% (0–10%) from old-age pensioners’ homes.

95% of the general rehabilitation facilities stated that they are informed about whether the patients are colonized or infected with MDRO prior to their admission (neurological early rehabilitation 100%). 4.5% of the general rehabilitation facilities stated that they always screened their patients upon admission (neurological early rehabilitation 100%), 64% at least sometimes, and only 18.2% reported no entrance screening at all (Table 2 (Tab. 2)). The rehabilitation facilities most often screened for MRSA (n=16; 72.7%), carbapenem-resistant Enterobacteriacae (CRE) (n=9; 40.9%), and 3MRGN (multidrug-resistant Gram-negative organisms, resistant against penicillins, cephalosporines, and fluorochinolones according to the definition of KRINKO, 2012) (n=8; 36.4%), and less often for VRE (n=6; 27.3%), ESBL (22.7%), and *Clostridioides *difficile (n=2; 9.1%), with higher screening rates in neurological early rehabilitation (Table 2 (Tab. 2)). All four early rehabilitation sections screened their patients for MRSA, three of them screened for VRE and CRE, and one facility also screened for *Clostridioides difficile*. 

Asked about the approximate percentage of patients colonized/infected with MDRO, general rehabilitation facilities estimated a mean prevalence of under 5%, and neurological rehabilitation units at almost 50% (for further details see Table 3 (Tab. 3)).

Two (9.1%) of the rehabilitation facilities reported delayed admission of patients with MDRO, while six (27.3%) reported refusing to admit patients with MRSA and/or CRE (Table 4 (Tab. 4)). 

Regarding items on management of patients with MDRO, 17 answers were obtained, including 4 (23.5%) that create new single rooms, two (11.8%) that create separate areas on the ward only for MDRO patients and/or dedicate equipment for individual MDRO patients, and one (5.9%) that creates separate sections for MDRO patients in therapy areas and/or dedicates equipment for MDRO patients (Table 4 (Tab. 4)). 

In order to prevent the spread of MDRO, the patients’ activities are restricted to therapy in the patient’s room and/or in a gym dedicated to MDRO patients (n=9; 40.9%), meals are served only in the patient’s room and attending hydrotherapy is not allowed (n=6; 27.3%). The patients are rarely allowed to participate in group therapy and social gatherings (n=3; 13.6%). Only six (27.3%) of the rehabilitation facilities stated that patients with MDRO are allowed to partake in a full rehabilitation program (Table 4 (Tab. 4)). 

Twenty facilities answered the question regarding the impact of isolating patients with MDRO on their rehabilitation outcome: most of them felt that the outcome was severely or moderately limited, only one facility (5%) stated that MDRO status does not limit the outcome of the patient (Table 5 (Tab. 5)). 

All institutions but one offer education or training on MDRO to doctors, nurses and other therapy staff, 19 to other ward staff (cleaning, housekeeping), 12 to the patients, and 9 to relatives and visitors (Table 6 (Tab. 6)).

## Discussion

Before discussing the results, some limitations must be mentioned: only 22 out of the 43 rehabilitation clinics in the region answered (response rate 58%). Although percentages should not be used in such small entities, we reported the results in numbers and percentages for better comparison with the data of Doherty et al. [22]. Regarding the specialization of the answering clinics, there were twelve orthopaedic, five cardiology, four neurology, four internal medicine, three psychosomatic, three pulmonology, two geriatric, two rheumatic, one paediatric and one urology unit, including four units for early neurological rehabilitation (multiple responses were possible). 

Since no non-responder analysis was conducted, a response bias cannot be ruled out; however, there is no evidence of a bias. The data were obtained anonymously, so it cannot be directly compared with the MDRO prevalence study carried out in the Rhine-Main region immediately preceeding ours [16]. 

### Hygiene structure 

Overall, a good hygiene structure (guidelines, hygiene personnel) was found in the facilities in the Rhine-Main area. More than 95% of the institutions stated that they used the KRINKO's national recommendations and that they were “always” or “sometimes” informed about the MDRO status before admission. This indicates almost complete compliance with the Hessian Hygiene Ordinance’s [25] requirement that the subsequent care facility must be informed in advance.

### Screening

Two-thirds of the facilities in the Rhine-Main area screened their patients sometimes, less than 20% never, and only one facility (4.5%) screened all patients. Screening was most common for MRSA (73%), and less often for 3MRGN or 4MRGN (approx. 40%). All four institutions for early neurological rehabilitation indicated screening all their patients for MRSA, and three of them also screen for VRE and MRGN.

KRINKO recommends screening for MRSA in all patients who had an inpatient hospital stay (>3 days) in the previous 12 months, all patients with a history of MRSA as well as with anamnestic contact with MRSA patients, all dialysis patients, patients with chronic skin lesions, patients who need chronic care, and those who had antibiotic therapy in the past 6 months or a catheter [19]. In two prevalence studies in 2014 and 2019 in the Rhine-Main area [14], [16], two-thirds of the patients had a medical history involving direct transfer from an acute care clinic or hospital stay in the last 6 months, and would thus have to be screened for MRSA solely based on this criterion. Other screening reasons were significantly less common (dialysis 0.3%, skin problems approx. 10%, care requirements and catheter approx. 10%). A re-evaluation of the data from the large survey in 2014 with more than 2,000 rehabilitation patients showed that the MRSA prevalence was identical for patients with and without a history of hospitalization (0.7% each) [14]. Hence, prior hospital stay was not a risk factor for MRSA colonization. Therefore, this screening indication for rehabilitation facilities can be questioned, and screening could be limited to patients with risk factors such as injuries of the skin and wearing medical devices. However, it makes sense to screen every patient in neurological early rehabilitation for MDRO.

The screening recommendations for 4MRGN include patients in contact with the health system in countries with endemic occurrence of 4MRGN in the last 12 months, patients in contact with patients with 4MRGN (care in the same room) and patients with hospitalization (>3 days) in a region (any country) with an increased 4MRGN prevalence [20]. Little can be deduced from our data in this regard, as only 1% of the patients stated that they had been hospitalized abroad, most of them in high-prevalence countries (2x Turkey, 1x each Spain, Greece, Italy, Bulgaria, Austria, France, and South Africa).

Regarding VRE, KRINKO recommends screening only as part of a bundled strategy in facilities where multiple VRE infections have occurred, not in all facilities [21]. In our study on the prevalence of VRE in 2019, 2.2% of 895 patients in general rehabilitation (orthopaedic, cardiologic, urologic etc.) and 33% of patients in early neurological rehabilitation exhibited VRE colonization; VRE infections did not occur in any facility [16].

### MDRO Prevalence

MDRO prevalence was estimated as follows: 0.3% MRSA, 0.9% VRE, and 2.5% MRGN (including 0.2% CRE), with significantly higher prevalence in the facilities for early neurological rehabilitation (1.4% MRSA; 20% VRE, 26% 3/4MRGN). Hence, the MDRO prevalence in general rehabilitation units was underestimated in comparison to the data of the prevalence surveys in 2014 and 2019 (0.7% MRSA, 2.2% VRE, 6.8% MRGN). In contrast, the prevalence estimated by the institutions for early neurological rehabilitation was comparable to the prevalence measured in 2019 (VRE 33%, 3MRGN 18%).

### Measures

Six rehabilitation centers refused to admit patients with MDRO and another two said that the admission of patients with MDRO was delayed. This clearly contradicts KRINKO's recommendations that the right to rehabilitation must not be infringed upon by MDRO colonization [19], [26].

When MDRO patients were admitted, the most common measures were cohorting, therapy with dedicated equipment, therapy in dedicated sections or at the end of the day. Almost half of the facilities performed therapies in the patient´s room or in special therapy rooms, more than a quarter of the facilities prohibited eating in the dining room and hydrotherapy, and more than 10% excluded MDRO patients from participating in group therapy or in social gatherings. Only a quarter of the facilities enabled unlimited participation in the full rehabilitation program.

In the KRINKO guidelines on MRSA [19], a medical risk analysis is required as a prerequisite for determining the hygiene measures in the respective facilities/departments. The risk analysis encompasses the colonization pressure in the facility (what is the prevalence?), risk factors for colonization/infection with MRSA (skin barrier injuries, e.g., wounds or decubiti; medical devices, e.g., catheters) and the risk of transmission (e.g., frequency of skin contact in the context of nursing care) to other patients. Based on this risk analysis, the best possible compromise should be achieved between preventing MRSA transmission and the possibility of participating in rehabilitation measures created by suitably designed processes. Thus, it is necessary to determine and document which rehabilitation measures deviating from the normal procedure may be carried out in a decentralized manner, e.g., in the patient's room (e.g., inhalation), and which ones must not be carried out at all (e.g., animal-assisted therapy). Basically, patients with MRSA colonization should be allowed to participate in rehabilitation measures, whereby the therapeutic devices and utensils used (balls, thermal packs, bathtubs, etc.) should be disinfectable and disinfected after use [19], [27], [28], [29].

In the prevalence study of the MDRO network Rhine-Main in 2014 [14], 0.7% of the patients in the general rehabilitation institutions exhibited MRSA colonization, 5–10% had wounds/decubiti and approx. 2% were supplied with catheters as risk factors for colonization/infection with MRSA; being bedridden as a risk factor for transmission in the context of nursing care was seen in 0.3% of the patients. As a result, neither high colonization pressure nor a high risk for transmission of MRSA was found in general rehabilitation. However, all risk factors were significantly higher in neurological and geriatric rehabilitation. Particularly in early neurological rehabilitation, these risk factors were present in almost all patients [16]. Intensified hygiene measures are therefore required in these departments.

With regard to MRGN, KRINKO recommends good basic hygiene in all wards regarding 3MRGN, but further hygiene measures up to isolation on at-risk stations; however, patients with 4MRGN should be isolated in all wards [20]. Transferring this recommendation to rehabilitation would result in an increased need for action for patients with 3MRGN in risk areas such as neurological (early) rehabilitation only. Advanced hygiene measures and possibly isolation would be required for patients with 4MRGN colonization or infection. According to our investigations, these patients still are very rare in rehabilitation (0.1%) [14], [16].

For VRE, KRINKO recommends compliance with good basic hygiene as long as there are no VRE infections requiring antibiotic therapy in a defined population, regardless of the number of colonized patients [21]. As infections with VRE were not reported from any of the rehabilitation facilities examined in 2019, not even from early neurological rehabilitation [16], according to KRINKO, no extended hygiene measures with regard to VRE are required in these facilities.

In the present survey, the KRINKO recommendations were obviously implemented and obeyed in many rehabilitation units. However, the general rejection of patients with MDRO reported by six institutions in no way complies with KRINKO guidelines. 

### Impact of isolation measures on the rehabilitation process

A quarter of the institutions assume that isolation of rehabilitation patients with MDRO will present a serious disadvantage for the outcome of the rehabilitation. Studies on the course of rehabilitation show that patients in early neurological rehabilitation with MDRO indeed have a worse outcome than patients without MDRO. A closer analysis revealed that this poorer rehabilitation outcome was essentially due to patients’ poorer state of health when being transferred from the acute clinic compared to patients without MDRO, whereas the improvement achieved during rehabilitation was comparable to that of patients without MDRO [30], [31], [32].

### Training

95% of the facilities offer MDRO training for doctors, nurses and therapists, but only 86% also include cleaning and household staff in the training. Advanced training in MDRO for patients and relatives was even less common, at 55% and 40%, respectively. However, the few MDRO patients and their relatives may be informed and trained in individual consultations as well.

### Comparison with the Europe-wide survey in 45 rehabilitation facilities

The results of our survey can be compared to the data from the Europe-wide survey in 45 rehabilitation facilities conducted in 2016, including nine institutions from Italy, four from Greece, two from Spain – more from high-prevalence countries – and only one from Germany [22]. Hence, it is plausible that the Europe-wide study indicates a much higher prevalence of MDRO. The comparability is further restricted, since only the total prevalence (MRSA, VRE, CRE, and ESBL) was queried in increments of 10%. Two-thirds of the European institutions estimated the prevalence below 10%, seven indicated 11-30% and six further 30-60%. The highest prevalence was reported from institutions in Greece, Italy, Spain and Turkey [22]. This distribution concurs with the resistance data of the ECDC from clinical materials in acute care hospitals in European countries [1].

The structures differed greatly between the rehabilitation facilities in our survey and the rehabilitation clinics in the European study. In the Rhine-Main region, 89% of all rehabilitation beds were offered in single-bed rooms and only occasionally in 3-bed rooms. In the European survey, 7% of the facilities had single rooms, 56% had double-bed rooms, 37% 3- to 4-bed rooms and 10% had rooms with more than 10 beds. Such multi-bed rooms complicate the management of patients with MDRO. The general recommendation for single rooms with attached sanitary modules in rehabilitation facilities promotes the rehabilitation of patients with MDRO. 

In the European study, 56% of the facilities encompassed less than 100 beds (9% had even less than 15 beds) and 42% of the facilities more than 100 beds. In the Rhine-Main area, only one facility offered fewer than 100 beds (1x80 beds), and 21 facilities had more than 100 beds (120–320 beds). The availability of guidelines for dealing with MDRO was significantly lower in the European study than in the institutions in the Rhine-Main area (80% vs. 100%).

The institutions of the European study reported screening for MDRO more frequently than did those in the Rhine-Main area (33% always and 38% sometimes), most often for MRSA (64%), followed by CRE (56%) and VRE (42%). This was comparable to our study (MRSA 73%, CRE 41%; VRE 27%).

One-third of the institutions in the European study stated that there were delays in the admission of patients with MDRO, and 11% refused to accept MDRO patients (Rhine-Main [RM] 9%, and 29%). In comparison with the data from the Rhine-Main area, the facilities from the European study took measures much more frequently to achieve single-bed rooms, separate areas and devices, and separate therapy times. This may be due to the different structures of the institutions. 36% (RM 45%) of the European institutions carried out therapies only in the patient’s room, 24% (RM 15%) refused to allow participation in group therapy and 20% in hydrotherapy; 16% urged MDRO patients to have their meals in their room and prohibited their participation in community events (RM 30% and 15%). 56% of the institutions in the European study stated that patients with MDRO were allowed to partake in the full rehabilitation program, compared to 27% in the Rhine-Main area. When patients with MDRO can be accommodated in single rooms, use only their own toilet, disinfect their hands before leaving their room and comply with good hand hygiene and general hygiene rules when coughing and sneezing, the risk of transmission is minimized and there should be no objection for them to use the dining room or therapy units. Of course, as KRINKO recommends, therapy equipment should be disinfectable and disinfected. 

Although the comparability of the two studies is limited, both studies still show many restrictions for rehabilitation patients with MDRO and thus a considerable need for improvement. The necessary hygiene recommendations to avoid transmission of MDRO must not lead to the refusal of inpatient rehabilitation or to less intensive rehabilitation. Pragmatic steps appropriate to each facility should be undertaken to ensure unlimited access of patients with MDRO to all rehabilitation measures in order to minimize any negative impact of their MDRO status on their rehabilitation outcome [22]. In Germany, many MDRO networks – including our MDRO network Rhine-Main as well – are working to improve rehabilitation for patients with MDRO, based on the specific recommendations of KRINKO.

## Notes

### Competing interests

The authors declare that they have no competing interests.

## Supplementary Material

Questionnaire: Management of multi-drug resistant organisms in rehabilitation facilities

## Figures and Tables

**Table 1:**
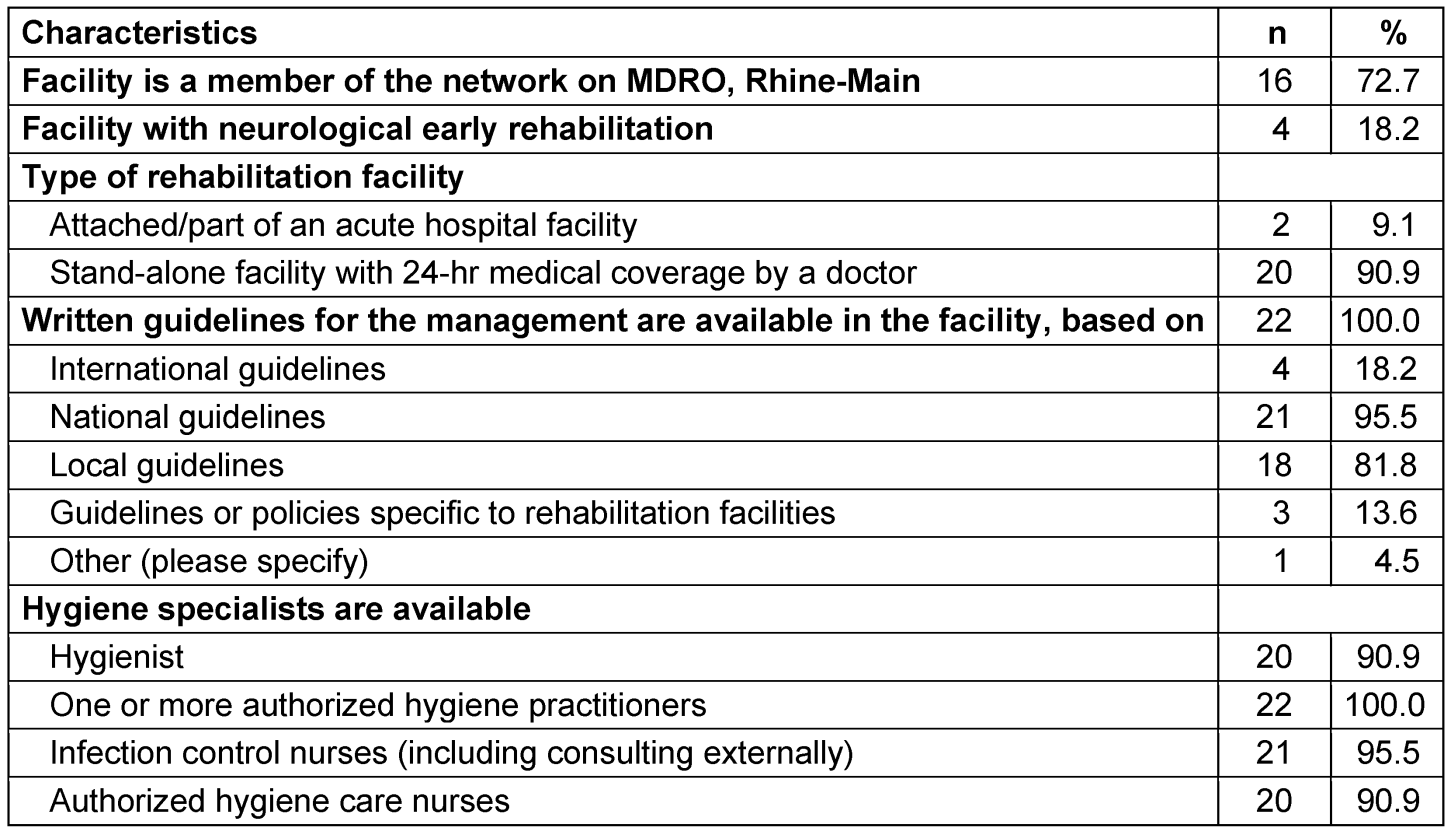
Stucture of the participating rehabilitation facilities (n=22)

**Table 2:**
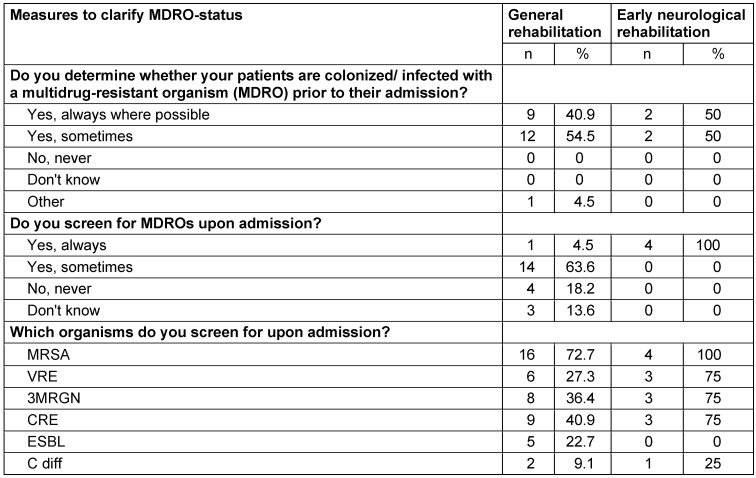
MDRO-status and Screening in 22 facilities for general rehabilitation and in four facilities for early neurological rehabilitation

**Table 3:**
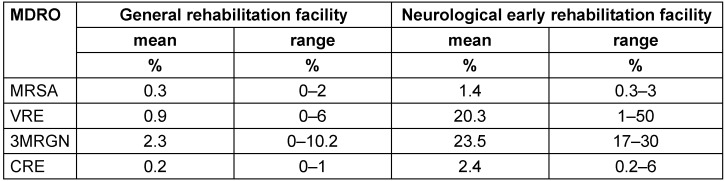
Prevalence of patients colonized/infected with MDRO in the 22 rehabilitation facilities (approximate data)

**Table 4:**
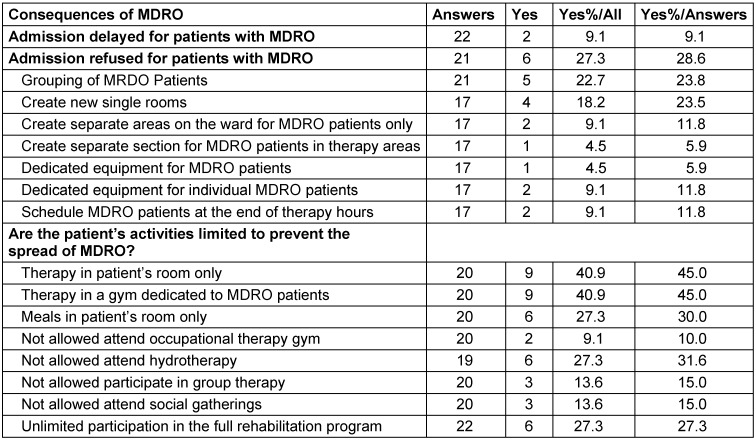
Impact of the measures on patients with MDRO Consequences for patients with MDRO in 22 rehabilitation facilities

**Table 5:**
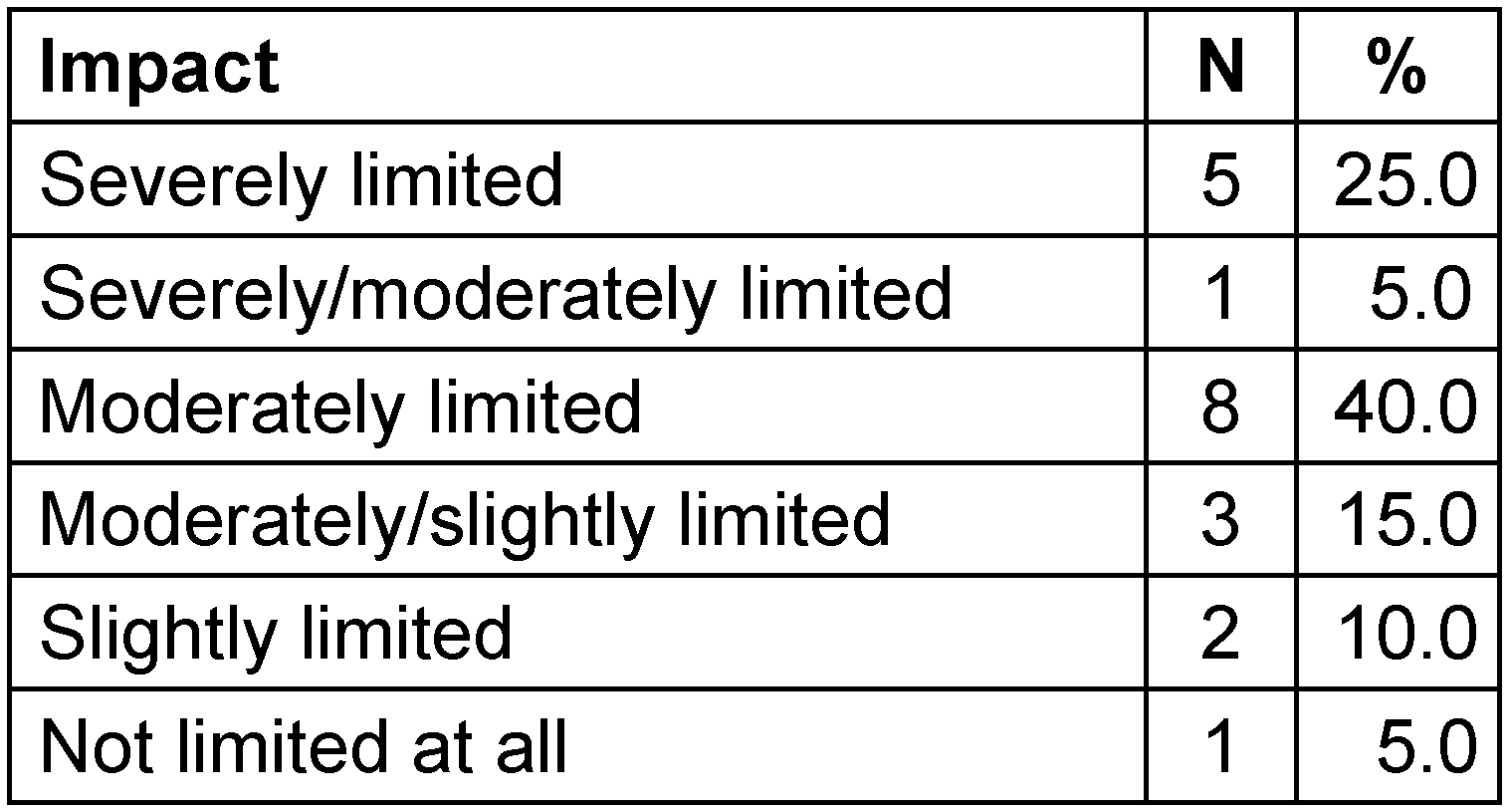
Impact of isolating patients with MDRO on their outcome

**Table 6:**
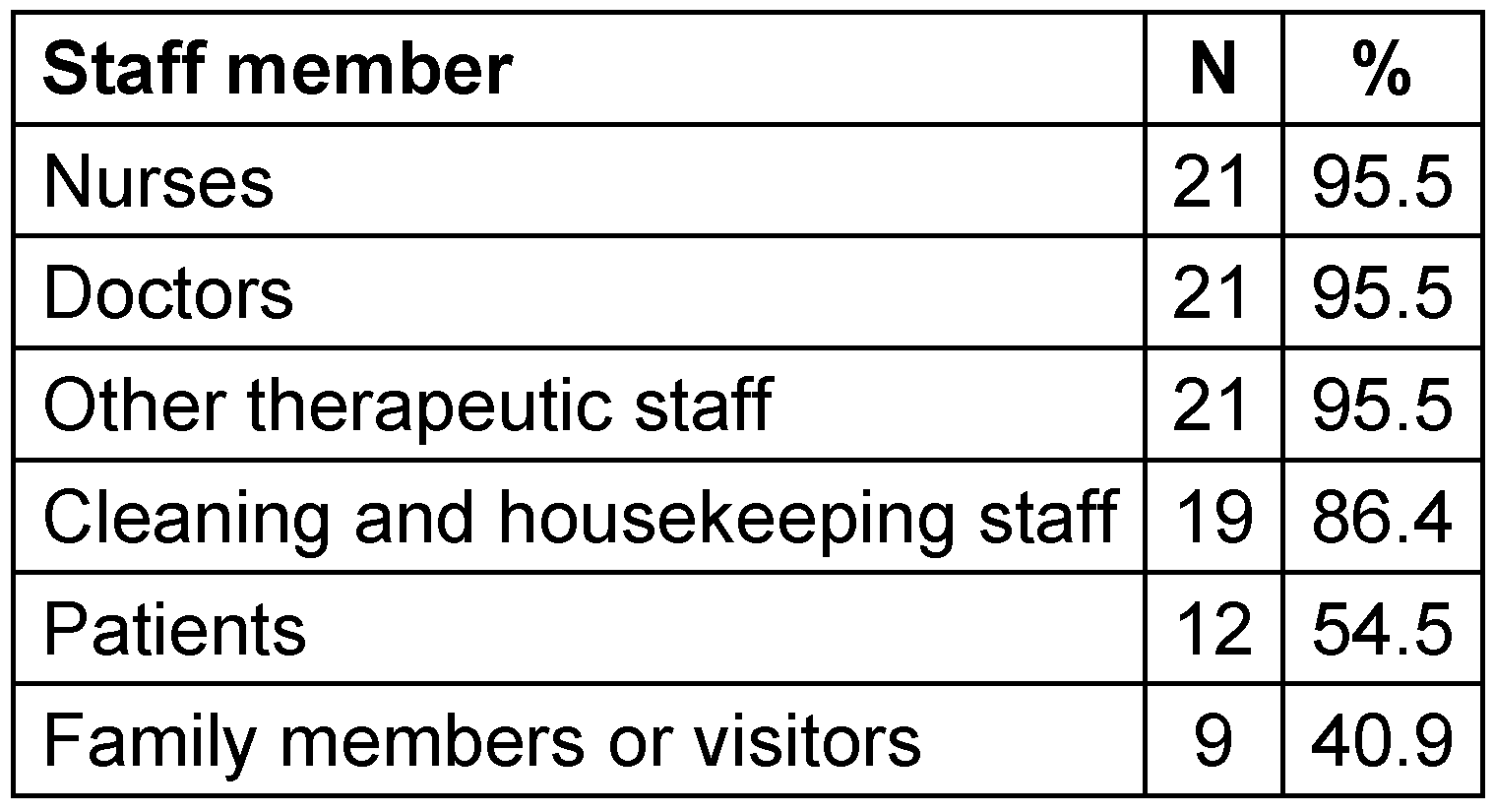
Offer of training and education on MDRO for the staff
