# Supplementary material for: Management of patients with multidrug-resistant organisms in rehabilitation facilities. Results of a survey in the Rhine-Main region, Germany, 2019
Source: GMS Hyg Infect Control. 2020 Jul 3;15:Doc15. doi: 10.3205/dgkh000350 (PMC7336045; doi:10.3205/dgkh000350)
Supplement: Questionnaire: Management of multi-drug resistant organisms in rehabilitation facilities [file HIC-15-15-s-001.pdf]

Attachment 1: Questionnaire

Management of Multi-Drug Resistant Organisms in Rehabilitation Facilities

Is your facility a member in the MDRO network Rhine-Main? *Yes O No O*

Main focus of your facility: *……………………………………………………………………………………*

Is there an early neurologic rehabilitation section in your facility? *Yes O No O*
(if yes: please answer questions page 2 and page 3 separately for general rehabilitation and early neurological rehabilitation; if no: please ignore all questions regarding early neurological rehabilitation)

What type of rehabilitation facility do you work in?

Attached/part of an acute hospital facility O

Stand-alone facility with doctor input on request only O

Stand-alone facility with 24-hr medical coverage by a doctor O

Other (please specify) ………………………………………………………………………………..

Does your rehabilitation facility follow a written guideline or policy on the management of patients with MDROs? Yes O No O

Which of the following policies/guidelines best describe the ones used in your facility?

International guidelines O

National guidelines *(i.e. KRINKO)* O

Local /*regional* guidelines (Guidelines of the MDRO network) O

Guidelines or policies specific to rehabilitation facilities O

Other (please specify)……………………………………………………………………………..

Are there hygiene specialists available at your facility?

Specialist in hospital hygiene (Physician) Yes O No O

Authorized hygiene practitioner (one or more) Yes O No O

Infection control nurse Yes O No O

Authorized hygiene-care nurses Yes O No O

How many rehabilitation beds do you have at your institution? …………….

How many single rooms? …………….

How many shared rooms? …………….

Regarding shared rooms: how many persons share one sanitary module? …………….

What proportion of your patients arrive from the following facilities?

Acute Care hospital ………% from their home ………%

Long-term care facilities for the elderly ………% other long-term care facilities………%

Attachment 1 to: Heudorf U, Berres M, Hofmann S, Steul K. Management of patients with multidrug-resistant organisms in rehabilitation facilities. Results of a survey in the Rhine-Main region, Germany, 2019. GMS Hyg Infect Control. 2020;15:Doc15.
DOI: 10.3205/dgkh000350

Please answer the following questions separately for general rehabilitation (GR) and – if available – for early neurological rehabilitation (ENR)

Do you establish whether your patients are colonized/infected with a multidrug-resistant organism (MDRO) prior to their admission?

General rehabilitation		Early neurological rehabilitation	
Yes, always where possible	O	Yes, always where possible	O
Yes, sometimes	O	Yes, sometimes	O
No, never	O	No, never	O
Don't know	O	Don't know	O
Other (please specify) ……………		Other (please specify) …………..	

Do you screen for MDROs upon admission?

General rehabilitation		Early neurological rehabilitation	
Yes, always where possible	O	Yes, always where possible	O
Yes, sometimes	O	Yes, sometimes	O
No, never	O	No, never	O
Don't know	O	Don't know	O
Other (please specify) ……………		Other (please specify) …………..	

If only some patients are screened, what percentage of patients do you screen?

General rehabilitation ………. % **Early neurological rehabilitation ……. %**

If only some patients are screened, which types of patients are selected for screening?

	GR	ENR
Those admitted from another institution	O	O
Patients who have previously tested positive for a multidrug-resistant organism (MDRO)	O	O
Only patients who will share a room with other patients	O	O
Don't know	O	O

Which organisms do you screen for on admission?

General rehabilitation		Early neurological rehabilitation	
MRSA	O	MRSA	O
VRE	O	VRE	O
3MRGN	O	3MRGN	O
4MRGN/CRE	O	4MRGN/CRE	O
ESBL	O	ESBL	O
Clostr. difficile	O	Clostr. difficile	O

Other (please specify) ……………………………………………………………

Attachment 1 to: Heudorf U, Berres M, Hofmann S, Steul K. Management of patients with multidrug-resistant organisms in rehabilitation facilities. Results of a survey in the Rhine-Main region, Germany, 2019. GMS Hyg Infect Control. 2020;15:Doc15.
DOI: 10.3205/dgkh000350

Are patients screened for MDROs prior to their discharge?

**General rehabilitation**		**Early neurological rehabilitation**	
Yes	O	Yes	O
No	O	No	O
Don't know	O	Don't know	O
Sometimes in certain circumstances		Sometimes in certain circumstances	
(Please explain briefly below)		(Please explain briefly below)	
………………………………………….		………………………………………..	

Approximately what percentage of your rehabilitation in-patients are colonized/infected with an MDRO?

**General rehabilitation**		**Early neurological rehabilitation**	
MRSA	……%	MRSA	……%
VRE	……%	VRE	……%
3MRGN	……%	3MRGN	……%
4MRGN/CRE	……%	4MRGN/CRE	……%
Don't know	O	Don't know	O

Do patients with MDROs have to wait longer for admission to your rehabilitation hospital?

Yes O   No O   Don't know O

Which MDROs were responsible for delaying admission?

MRSA	O
VRE	O
3MRGN	O
4MRGN/CRE	O
ESBL	O

Other (please specify) ……………………………………………………………

Have patients been refused entry to your rehabilitation facility on the basis of their MDRO status?

Yes O   No O   Don't know O

Are patients with MDROs grouped in separate wards or ward areas?

Yes O   No O   Don't know O

If the previous question was answered with yes: How are the patients grouped?

Create new single rooms	O
Create separate areas on the ward for MDRO patients only	O
Create separate section for MDRO patients in therapy areas	O
Dedicate equipment for MDRO patients	O
Dedicate equipment for individual MDRO patients	O
Schedule MDRO patients at the end of therapy hours	O
Don't know	O

Other (please specify)……………………………………………………………………………

Have you altered your rehabilitation facilities in any way to accommodate MDRO patients?

Yes O No O Don't know O

Are the activities of patients with MDROs limited in any of the following ways to prevent the spread of these organisms?

Therapy in patient’s room only	O
Therapy in a gym dedicated to MDRO patients	O
Meals in patient’s room only	O
Not allowed to attend physiotherapy gym	O
Not allowed to attend occupational therapy gym	O
Not allowed to attend hydrotherapy	O
Not allowed to participate in group therapy	O
Not allowed to attend social gatherings	O
Don't know	O

Other (please specify)…………………………………………………………………….

Are patients with MDROs allowed to participate in a full rehabilitation program?

Yes O No O

What impact do you feel isolating patients with MDROs has on their outcome?

Severely limits outcome	O
Moderately limits outcome	O
Slightly limits outcome	O
Does not limit outcome	O

Other (please specify)……………………………………………………………………

To which of the following groups, if any, does your institution offer education or training in relation to MDROs? (Please select all that apply)

Nurses	O
Doctors	O
Allied Health Professionals	O
Other Ward Staff	O
Patients	O
Family members or visitors	O
Others	O
No education or training relating to MDROs is provided	O
Don't know	O

Thank you very much!

The questionnaire was adopted from Doherty A, McNicholas S, Burger H, Boldrini P, Delargy M. European survey of management of patients with multidrug-resistant organisms in rehabilitation facilities. Eur J Phys Rehabil Med. 2019 Aug;55(4):418-423. DOI: 10.23736/S1973-9087.19.05570-9
